# Gut Microbiota Signatures in Gestational Anemia

**DOI:** 10.3389/fcimb.2021.549678

**Published:** 2021-02-25

**Authors:** Yan Long, Fang Liang, Ruochun Guo, Chunyan Zhu, Xueqin Zhao, Xifan Wang, Fei Liu, Min Jiang, Qihua Liang, Shanshui Zeng, Mengru Han, Junjie Qin, Shenghui Li, Shaochuan Li, Hongling Yang

**Affiliations:** ^1^ Department of Laboratory, Guangzhou Women and Children’s Medical Centre, Guangzhou Medical University, Guangzhou, China; ^2^ Shenzhen Promegene Technology Co., Ltd, Shenzhen, China; ^3^ School of Public Health, Guangzhou Medical University, Guangzhou, China; ^4^ School of Biology and Biological Engineering, South China University of Technology, Guangzhou, China

**Keywords:** gut microbiota, microbial dysbiosis, 16S rRNA gene sequencing, pregnant women, gestational anemia

## Abstract

Gestational diseases are associated with altered intestinal microbiota in pregnant women. Characterizing the gut microbiota of gestational anemia (GA) may describe a novel role of gut microbial abnormality in GA. In this study, we investigated differences in gut microbiota between GA patients and healthy pregnant women from the first trimester (n = 24 *vs.* 54) and the third trimester (n = 30 *vs.* 56) based on the 16S rRNA gene sequencing method. No statistically significant differences in α-diversity were identified between GA patients and controls in the first trimester of pregnancy, whereas the Shannon index and observed OTUs were significantly lower in GA patients than in healthy controls in the third trimester. Distance-based redundancy analysis revealed striking differences in microbial communities in the third trimester between GA patients and controls. Four genera were significantly different in relative abundance between GA patients and healthy controls, while 12 genera differentiated significantly between GA patients and healthy controls in the third trimester. At the operational taxonomic unit (OTU) level, 17 OTUs and 30 OTUs were identified to be different between GA patients and healthy controls in the first and third trimesters, respectively. Changes in gut microbial composition of GA patients suggest a potential relation with GA, and provide insights into the prediction and intervention of gestational anemia.

## Introduction

Anemia remains a major health problem worldwide, especially in third-world countries (Milman, 2011). The global anemia prevalence was estimated to be approximately 33% in 2010 (Kassebaum et al., 2014). Specifically, gestational anemia (GA), which occurs in pregnant women, is one of the most common types of anemia (W. H. Organization, 1992). According to the World Health Organization, the prevalence of GA was more than 40% in 2011 (World Health Organization, 2019). A cross-sectional study showed that GA prevalence reached 58.6% in the third trimester of pregnancy for women in China (Ma et al., 2009). Anemia in pregnancy can impose a heavy physiological and economic burden on patients. GA may significantly affect the health status of both mothers and their fetuses (Ma et al., 2009). Previous studies have indicated that gestational anemia may increase the risk of intrauterine growth restriction, preterm birth, and low birth weight (Katz et al., 2006; Lee et al., 2006). Severe anemia can also increase perinatal morbidity (Prema et al., 1981) and lead to maternal death (Starrs, 2015).

There are a variety of causes of anemia. Nutritional deficiency of iron may result in development of iron deficiency anemia (IDA), which leads to a total burden of about 75 to 80% of anemia cases (Milman, 2011). Similarity, a lack of nutritional elements such as folate (Al Khatib et al., 2006; de Benoist, 2008), vitamin B12 (Pinto et al., 1973), and vitamin D (Özsoylu and Aytekin, 2011) can also contribute to anemia. Moreover, microbe invasion is also another important pathogenic factor for anemia; for example, malaria parasite can lead to sequestration in the placental vascular space with consequent maternal anemia (Shulman et al., 1996; Cot et al., 1998).

In recent years, there has been emerging evidence indicating the role of gut microbiota in human nutrition and metabolism. Studies in iron-deficient women from India indicated that the intestinal microbiota of individuals with IDA are relatively deficient in *lactobacillus* (Balamurugan et al., 2010). Another study reported gut microbiological disorders in infants and young children with nutritional IDA (Muleviciene et al., 2018), based on 16S analyses of fecal samples. Moreover, gut microbiota can promote hematopoiesis at primary immune sites of the host (Khosravi et al., 2014), indicating the essential role of the gut microbiome in anemia. In addition to human nutrition, a study revealed that changes of gut microbiota may impact the circulating levels of short-chain fatty acids (SCFAs), further influence bone marrow hematopoiesis and the progress of infection (Jaeggi et al., 2015).

Pregnant women with GA comprise one of the most vulnerable cohorts of patients suffering from anemia. However, the alteration of gut microbiota and the association between the altered bacterial taxa and GA disease in pregnant women remains poorly understood. In this study, we explored the characteristics of gut microbial composition in 54 GA pregnant women in the first and third trimesters compared to 110 age- and body-weight-matched controls using 16S rRNA gene sequencing.

## Materials and Methods

### Ethics Statement

This study received approval from the Ethics Committee of Guangzhou Women and Children’s Medical Centre, and informed consent was obtained from each subject. These methods were carried out in accordance with the Declaration of Helsinki (World Medical Association Declaration of Helsinki, 2014).

### Study Design and Fecal Sample Collection

The matched case-control study recruited participants from Guangzhou Women and Children’s Medical Centre from January 2017 to December 2017. Enrolled patients included 24 women with gestational anemia (GA1 group) and 54 gender-, age-, and body-weight-matched healthy pregnant women (HC1 group) in the first trimester, as well as 30 women with gestational anemia (GA3 group) and 56 healthy controls (HC3 group) in the third trimester. In the present study, the inclusive criterion for gestational anemia was considered to be serum hemoglobin (HGB) ≤105 g/L, while the healthy controls were defined to be HGB ≥120 g/L. The phenotypic characteristics of all participants are summarized in **Table 1**. All participants were Chinese and no participants with alcoholism, smoking, strict vegetarians, or with other unusual dietary habits during pregnancy. The exclusion criteria included participants who had taken antibiotic treatment or probiotic supplements in the 4 weeks prior to sample collection. Baseline information was measured and fecal samples were collected by well-trained staff, strictly following standard procedures. Stool samples were put into a foam box filled with ice packs and transported to the laboratories as quickly as possible. Samples were stored at −80°C until DNA extraction and 16S rRNA gene sequencing. For all participants, iron supplementation intaking condition was conducted by questionnaire. No significant difference in iron supplement intake between GA group and HC group.

**Table 1:** Characteristics of the subjects.

		GA patients	Healthy controls	*P*-value
	No. of samples	24	54	
**1st trimester**	HGB (g/L)	97.8 ± 7.5	128.8 ± 5.8	<0.001
	Age (year)	28.8 ± 3.6	30.0 ± 3.4	0.189
	PBMI (kg/m^2^)	19.7 ± 2.1	19.9 ± 1.6	0.667
	Gestational days	100.9 ± 6.5	102.3 ± 7.0	0.409
	No. of samples	30	56	
**3rd trimester**	HGB (g/L)	99.0 ± 4.7	125.9 ± 5.8	<0.001
	Age (year)	31.2 ± 3.9	30.8 ± 3.6	0.624
	PBMI (kg/m^2^)	20.3 ± 2.0	20.6 ± 2.1	0.410
	Gestational days	236.8 ± 8.0	235.6 ± 7.9	0.513

The data for GA patients and healthy controls are presented as the mean ± SD. P-values were calculated by Student’s t-test. HGB, hemoglobin; PBMI, pre-pregnant body mass index.

### DNA Extract and Sequencing

Microbial DNA was extracted from stool samples collected by subjects in the hospital according to the MOBIO Power Soil ^®^DNA Isolation Kit 12888-100 protocol. All of the DNA was stored in a freezer at −80°C before sequencing. Designed unique fusion primers with universal primers set, 515F (5′ -GTGYCAGCMGCCGCGGTAA-3′) and 806R (5′ -GGACTACNVGGGTWTCTAAT-3′), along with barcode sequence were used to amplify the V4 region of 16S rRNA gene. PCR mixtures and thermal cycling were performed as previously described (Li et al., 2019). Amplicons from each sample were run on an agarose gel. The expected band size is ~300–350 bp. Amplicons were quantified with the Quant-iT PicoGreen dsDNA Assay Kit (ThermoFisher/Invitrogen cat. no. P11496; following the manufacturer’s instructions).

The amplicon library was pooled in equal amount and subsequently quantified (KAPA Library Quantification Kit KK4824). Then paired-end sequencing on Illumina MiniSeq platform at Promegene Co. Ltd (Shenzhen, China) was performed. Read length is 150 bp excluding the primer sequences.

### Bioinformatic Analyses

Raw sequencing reads, which produced >8 homopolymers, >2 mismatches in the primers, or >1 mismatches in the barcode, were removed in pairs. High-quality sequencing reads were analyzed *via* the quantitative insights into microbial ecology (QIIME2, https://qiime2.org/) platform (Kuczynski et al., 2012) and the standard tools/plugins provided by QIIME2. Briefly, the 16S sequences were analyzed for further quality control and to feature table construction using the DADA2 algorithm (Callahan et al., 2016). The remaining reads were truncated from 0 to 140 bases (for both forward and reverse reads) to avoid sequencing errors at the end of the reads. Paired-end reads were overlapped at the maximum mismatch of six bases, which created a minimum similarity threshold of approximately 90% on the overlap zone of the forward and reverse reads. The representative sequences (named “feature” in QIIME2 nomenclature) were then generated by removing the redundant and low occurrence (n < 5 in pool samples) sequences. We used the term “operational taxonomic unit (OTU)” instead of “feature” throughout this article for convenience. Then, taxonomic assignment of the OTUs was determined based on a pretrained Naive Bayes classifier (DeSantis et al., 2006) (trained on the Greengenes 13_8 99% OTUs) *via* the q2-feature-classifier plugin. The taxonomic compositions at the phylum, class, order, family, genus, and species levels were generated based on OTU annotation. To avoid sampling depth bias, 20,000 reads were randomly selected from each sample when calculating the OTU and taxa relative abundances.

Phylogenetic analyses were implemented *via* the q2-phylogeny plugin, which performed multiple sequence alignments on the OTU sequences and generated phylogenetic trees of the OTUs from the alignment results. Four estimators of the α-diversity, including Shannon’s diversity index, observed OTUs, Faith’s phylogenetic diversity (a qualitative measure of community richness that incorporates the phylogenetic relationships between the OTUs) and Pielou’s evenness, and Bray-Curtis dissimilarity (an estimator of the β-diversity) was used in this study and calculated based on the QIIME2 q2-diversity plugin.

### Statistical Analyses

Statistical analyses were implemented using the R platform. Distance-based redundancy analysis (dbRDA) was performed on normalized taxa abundance matrices with R vegan package (Dixon, 2003) according to Bray-Curtis dissimilarity, and then visualized with R ggplot2 package. The disease-associated genera, OTUs, and taxa were identified based on the Wilcoxon rank-sum test. Random forest models were trained with R randomForest package (10,000 trees) to predict disease status according to OTU and genus abundance profiles. The performance of the predictive model was evaluated with leave-one-out cross-validation method. Receiver operator characteristic (ROC) analysis was performed using R pROC package. A P-value of <0.05 was considered statistically significant, and the q-value was calculated to evaluate the false discovery rate for correction of multiple comparisons.

## Results

### Study Cohort and Sequencing Data

To investigate the associations between the gut microbial composition and gestational anemia in pregnancy, we analyzed the fecal samples of healthy controls and women with GA in the first (n = 24 *vs.* 54) and third (n = 30 *vs.* 56) trimesters. According with the meta-analysis (Smith et al., 2019; Rahmati et al., 2020), the first and the third trimester have greater impact on pregnancy outcome, for example, anemia at the first and the third trimesters is associated with preterm birth and low birth weight. Therefore, we enrolled the pregnant women in the first trimester and the third trimester, which refers to the method of sample collection in some articles (Koren et al., 2012; Nuriel-Ohayon et al., 2019). Patients and controls were matched according three indicators (age, BMI, and gestational age) in each trimester (**Table 1**), and the first and third trimester cohorts are independent of each other without any overlap. Basic information of participants is shown at **Table 1**.

The gut microbiota from 164 fecal samples was profiled using high throughput 16S rRNA gene sequencing of the V4 variable region. A total of 8,539,948 high quality sequences (52,073 ± 11,821 sequences per sample) were ultimately produced. And 2,453 OTUs were identified and functionally labeled using QIIME2 platform, as previously discussed.

### Reduced Diversity in Gestational Anemia in the Third Trimester

**Figure 1:**
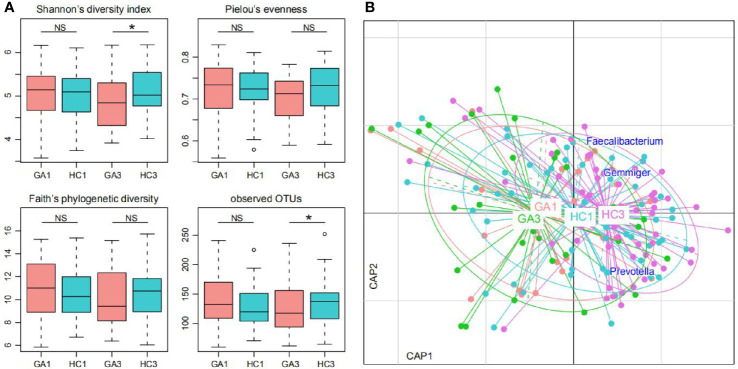
Difference of gut microbial community between GA patients and controls. **(A)** Difference of α-diversity between GA patients and controls. Significance levels in correlation tests are denoted: NS, P > 0.05; *P ≤ 0.05. **(B)** dbRDA based on the Bray–Curtis dissimilarity between microbial genera, revealing GA microbial dysbiosis, which overlaps only in part with taxonomic composition in patients and controls. Patient and control samples were mainly separated in the primary constrained axis. Lines connect samples (colored points) in the same group, and circles cover samples near the center of gravity for each group. Genera (blue squares) as the main contributors are plotted by their loadings in these two components.

The microbial α- and β-diversity indices were used to evaluate the richness and inter-sample relationships of gut microbiota of all participants. No statistically significant differences in α-diversity were found between patients and controls in the first trimester of pregnancy (**Figure 1A**). However, when we focused on the third trimester of pregnancy, Shannon’s diversity index and observed OTUs of gut microbiota in the GA group were significantly lower than those in the control group. The other three indices—Pielou’s evenness, Faith’s phylogenetic diversity index, and the observed OTUs diversity index—were lower in tendency. Furthermore, distance-based redundancy analysis (dbRDA) based on Bray–Curtis dissimilarity between microbial genera captured visible separation of GA stratification on the intestinal microbiota when compared to the control group in both of the two time periods. However, the differences were not significant (Adonis p > 0.05) (**Figure 1B**). On the dbRDA plot, GA acted on the primary and the second constrained axis (17.7% variance explained), while *Prevotella*, *Faecalibacterium*, and *Gemmiger* were the major contributors.

### Comparison of the Gut Microbiota

**Figure 2:**
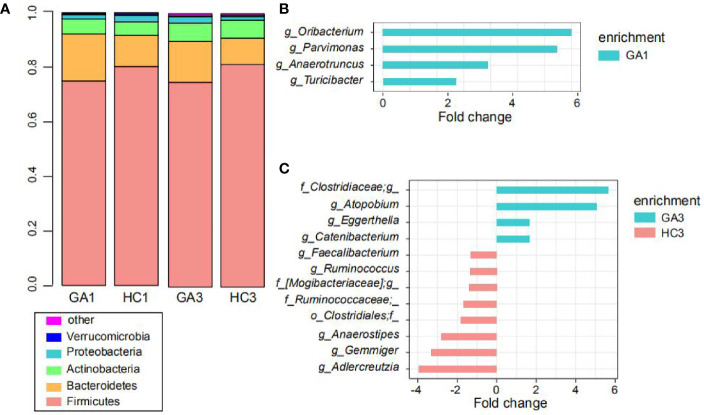
Comparison of the gut microbiota. **(A)** Top five most abundant bacterial phyla in the GA patients and healthy controls in the first and third trimesters. Each colored box represents a bacterial taxon and the height of a colored box represents the relative abundance of that organism within the sample. Bacteria ranked below fifth, as well as unclassified phyla, are grouped as “other.” **(B, C)** Bacterial genera showing significant differences in relative abundance between the GA patients and healthy controls in the first **(B)** and third **(C)** trimesters.

At the phylum level, the predominant sequences in the GA1 were from *Firmicutes* (75%), *Bacteroidetes* (17.2%), *Actinobacteria* (5.5%), *Proteobacteria* (1.6%), and *Verrucomicrobia* (0.4%), while the sequences for the HC1 group belonged to *Firmicutes* (80.3%), *Bacteroidetes* (11.4%), *Actinobacteria* (5%), *Proteobacteria* (2.4%), and *Verrucomicrobia* (0.6%). These five phyla comprised more than 99% of the relative abundance in the two groups (**Figure 2A**). In the third trimester of pregnancy, *Firmicutes* was still the dominant phylum, which accounts for 75% abundance in GA3 and 81.4% in HC3; *Bacteroidetes*, *Actinobacteria*, *Proteobacteria*, and *Euryarchaeota*, contributed to 15, 6.7, 2.4, and 0.2% abundance of the GA3 group and 9.7, 6.6, 1.4, and 0.4% abundance of the HC3 group, respectively (**Figure 2A**). The ratio of *Firmicutes/Bacteroidetes* in GA patients compared to controls expanded from 4.36 to 7.04 in the first trimester and 5.0 to 8.39 in the third trimester.

At the genus level, *Turicibacter*, *Oribacterium*, *Parvimonas*, and *Anaerotruncus* were significantly enriched in the GA1 group compared to the HC1 group (**Figure 2B**), whereas no genus was significantly enriched in HC1. In addition, eight genera, including *Adlercreutzia*, *Gemmiger*, *Anaerostipes*, Ruminococcaceae^*^, *Clostridiales* ^*^, [Mogibacteriaceae]^*^, *Ruminococcus*, and *Faecalibacterium* were more represented in the HC3 group (**Figure 2C**), while *Atopobium*, Clostridiaceae^*^, *Catenibacterium*, and *Eggerthella* were enriched in the GA3 group compared to HC3 group.

**Figure 3:**
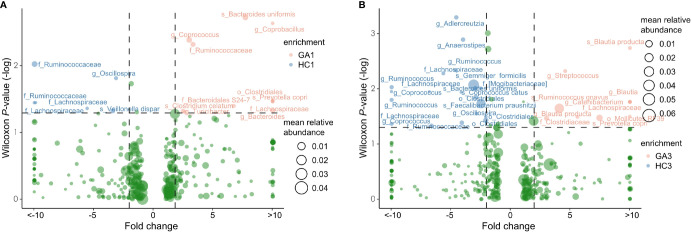
Volcano plot showing significant variation in OTUs between patients and controls in the first **(A)** and third **(B)** trimesters. Only the OTUs with average relative abundances greater than 0.01% of total abundance in all samples are shown for clarity. Red and blue circles represent the GA- and control-enriched OTUs, respectively.

After abundance filtering at an average relative abundance threshold of 0.01%, 483 OTUs and 497 OTUs were obtained in the first and third trimesters, respectively. Six OTUs, including two each from family *Ruminococcaceae*, family *Lachnospiraceae*, species *Veillonella dispar*, and genus *Oscillospira* were enriched in GA patients in the first trimester compared with healthy controls. There were 11 OTUs that were depleted in GA patients in the first trimester, including genus *Coprococcus*, species *Bacteroides uniformis*, family *Ruminococcaceae*, species *Clostridium celatum*, genus *Turicibacter*, genus *Bacteroides*, *o_Clostridiales*, family *Bacteroidales_ S24-7*, species *Prevotella copri*, genus *Coprobacillus*, and family *Lachnospiraceae* (**Figure 3A**). In the third trimester, 20 OTUs, such as species *Gemmiger formicilis*, species *Faecalibacterium prausnitzii*, species *Bacteroides uniformis*, species *Coprococcus catus*, genus *Anaerostipes*, genus *Ruminococcus*, genus *Adlercreutzia*, family *[Mogibacteriaceae]*, and family *Lachnospiraceae* were identified to be more abundant in pregnant women with anemia. In contrast, another 10 OTUs, including three from genus *Blautia*, species *[Ruminococcus] gnavus*, species *Prevotella copri*, family *Clostridiaceae*, genus *Streptococcus*, genus *Catenibacterium*, family *Lachnospiraceae*, and *o_Mollicutes_ RF39*, were enriched in pregnant women without anemia (**Figure 3B**). It is worth noting that genus *Oscillospira* were depleted in pregnant women without anemia in both trimesters.

### Gestational Anemia-Related Bacteria Are Associated With Inflammation and Metabolism

For GA-related bacteria in first trimester, family *Lachnospiraceae* and species *Prevotella_copri* are negatively correlated with liver metabolism index, such as γ-GT, TP, ALB, TBA, GLO, ALT, AST, HDP, and BIL. Genus *Coprococcus*, family *Lachnospiraceae*, species *Bacteroides uniformis*, and family *Ruminococcaceae* are negatively correlated with inflammation index, such as BA, PCT, WBC, and EO, while species *Prevotella copri* and genus *Coprococcus* are positively correlated with BA (**Figure S5A**).

For GA-related bacteria in third trimester, genus Ruminococcus, species Bacteroides uniformis, genus Anaerostipes, order Clostridiales, species Faecalibacterium_prausnitzii, species Gemmiger formicilis, species Coprococcus catus, genus Adlercreutzia, and family Lachnospiraceae are positively correlated with liver metabolism index (ALB and ALB/GLO), and inflammation index (WBC, MCH, and MCV), while species Ruminococcus gnavus and species Blautia producta are negatively correlated with liver ALB, ALB/GLO, MCH, and MCV BA (**Figure S5B**).

### Gut Microbiota-Based Classification of Gestational Anemia

**Figure 4:**
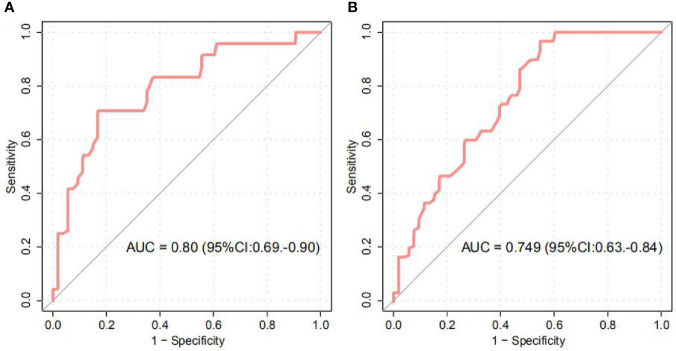
Random Forest Classification of GA status by the abundance of gut microbiota. **(A, B)** ROC analysis for classification of GA status in the first **(A)** and third **(B)** trimesters by significantly different OTUs, assessed by AUC.

Our study further provided we evaluated the ability of intestinal microbial composition to classify the anemic status of women. The models, trained from the abundance of OTU biomarkers in first and third trimesters, achieved the area under the ROC curve (AUC) of 0.80 (95% CI 0.69 to 0.90) and 0.75 (95% CI 0.63 to 0.84) for discriminating GA and healthy controls in the first or third trimester, respectively (**Figures 4A, B**, **S1**–**S3**). In addition, *Blautia*, *Odoribacter*, Ruminococcaceae^*^, *Turicibacter*, and Clostridiales^*^ featured the highest score in the model.

## Discussion

In the present study, we characterized the gut microbiota of pregnant women with anemia during at the first and third trimesters compared with those of healthy pregnant women. The results identified some differences in the composition of gut microbiota between anemia patients and normal controls.

In this study, although no significant differences in microbial α-diversity were found between GA patients and healthy controls in the first trimester, a significantly reduced α-diversity was found in GA patients during the third trimester. A reduction in α-diversity in gut microbiota has also been described in other diseases, including Crohn’s disease (Manichanh et al., 2006), atopic eczema (Wang et al., 2008), and myalgic encephalomyelitis/chronic fatigue syndrome (Giloteaux et al., 2016). The dbRDA analysis based on the Bray-Curtis distance (a β-diversity index) revealed that a common microbial feature emerged in our study. This indicates that the gut microbiota was significantly altered in GA patients. Changes in gut microbiota were reported to be associated with gestational diseases, such as gestational diabetes mellitus and early-onset preeclampsia (EOPE). Although the causes of gestational anemia remain elusive, our present study suggests that the composition of gut microbiota may be involved in the pathogenesis of GA.

The overall microbial composition of the fecal samples indicated that there were healthy adults in our study, with a dominance of taxa from the phyla of the *Firmicutes*, *Bacteroidetes*, and *Actinobacteria* (Curtis et al., 2012). It is worth noting that *Firmicutes* were less abundant in GA patients than in healthy controls, while *Bacteroidetes* were increased in GA patients compared to the healthy group in both trimesters. Moreover, the ratio of *Firmicutes/Bacteroidetes*, which may be positively correlated with obesity in healthy adults (Koliada et al., 2017), was higher in healthy controls than in GA patients. In agreement with this, thin women were more likely to be anemic than women of normal weight (Bentley and Griffiths, 2003).

At the genus level, *Faecalibacterium* was dominant in all four cohorts, and its abundance in GA patients was significantly lower than that in healthy controls in the third trimester. Consistently, at the OTU level, *F. prausnitzii*, which accounted for the majority of the abundance of *Faecalibacterium*, was also significantly decreased in GA patients in the third trimester. The importance of *F. prausnitzii* in the composition of gut microbiota has been demonstrated by many case control studies, such as colorectal cancer (Sobhani et al., 2011), liver transplantation (Wu et al., 2012), and chronic idiopathic diarrhea (Swidsinski et al., 2008). Decreased abundance of *F. prausnitzii* has been reported to be associated with dysbiosis caused from numerous disease (Miquel et al., 2013). For example, previous studies found that low levels of *F. prausnitzii* could be predictive for Crohn’s disease (Sokol et al., 2008), and another study indicated that patients with Celiac disease have a significant decrease in the relative abundance of *F. prausnitzii* (De Palma et al., 2010).

Similarly, the relative abundance of *Oscillospira* (F0167), which was thought to be negatively associated with inflammatory diseases and body mass index (Konikoff and Gophna, 2016), was significantly higher in the healthy group than in the GA group during both trimesters. *Oscillospira* was decreased in patients or relatively severe patients in two studies of inflammatory bowel disease (Santoru et al., 2017) and human immunodeficiency virus infection (Vesterbacka et al., 2017). Coincident results emerged in another genus of *Ruminococcus*, which was more abundant in healthy controls than in GA patients in the third trimester. It is not difficult to find that both *Faecalibacterium* and *Ruminococcus* are considered to be butyrate-producing members in human gut (Takahashi et al., 2016), and some of *Oscillospira* may also have the ability to produce butyrate (Gophna et al., 2017). Existing research indicated that butyrate plays a crucial role in the prevention of inflammation (Cushing et al., 2015) and metabolic diseases (Qin et al., 2012; Karlsson et al., 2013; Vrieze et al., 2014; Arora and Backhed, 2016).

Our study further provided the microbial markers for GA discrimination, and achieved AUC of 0.80 and 0.75 for identifying disease status in the first and third trimesters, respectively. Future systematic investigations of these OTU and gene markers that featured the highest score for the discrimination of disease status would be of value. However, to better explore a good potential for prediction and early diagnosis of GA from the fecal microbiota in the first trimester for the third trimester, a longitudinal study needs to be performed where fecal samples from early pregnancy (non-anemic at that time) of pregnant women who develop anemia at late pregnancy in order to avoid the two cohorts (first and third trimesters) are independent of each other.

This study is not without limitations. Although our samples were age- and BMI-matched, other environmental and behavioral factors, such as geography (Kumar et al., 2016), lifestyle (Conlon and Bird, 2014), diet (Paola et al., 2010), and drug usage (Panda et al., 2014) may have contributed to the gut microbial community of GA patients. Moreover, this was a cross-sectional study with a relatively small number of participants, and thus, large-scale cohort studies containing multiple types of GA are needed to confirm the results.

To our knowledge, this is the first study to explore the relationship between gut microbiota and gestational anemia in both the first and the third trimesters of pregnancy. We demonstrated that GA patients may suffer from microbiota dysbiosis, which is mainly manifested by lower diversity and changes in some microbial taxa compared with healthy controls. Our findings on potential biomarkers not only extend the knowledge of the etiology of anemia in pregnant women, but also provide insights into the prediction and intervention of anemia and gestational anemia.

## Data Availability Statement

The raw sequencing dataset analyzed in this study has been deposited in the European Bioinformatics Institute (EBI) database under the accession code PRJEB31743 (https://www.ebi.ac.uk/ena/data/view/PRJEB31743). The OTU and taxonomic composition data, and the statistical scripts are available from the corresponding authors upon reasonable request.

## Ethics Statement

The studies involving human participants were reviewed and approved by Ethics Committee of Guangdong Women and Children Hospital. The patients/participants provided their written informed consent to participate in this study.

## Author Contributions

HY, SHL, and SCL conceived and directed the study. FaL and RG did the analysis and visualization. XZ, FeL, MJ, QL, SZ, and MH collected the samples. YL and FaL drafted the manuscript. XW, CZ, and JQ revised the manuscript. All authors contributed to the article and approved the submitted version.

## Funding

This study was funded by the National Natural Science Foundation of China (NSFC31570116, 81871716), the Natural Science Fund of Guangdong Province (2018A0303130314), the Science and Technology Fund of Guangzhou (201707010019, 201707010182), and the Shenzhen Science and Technology Innovation Committee (CYZZ2017331174025983), and the financial support of National Key R&D Program of China (2017YFD0400301).

## Conflict of Interest

Authors SHL, FaL, RG, XW, and JQ were employed by the company Shenzhen Promegene Technology Co., Ltd.

The remaining authors declare that the research was conducted in the absence of any commercial or financial relationships that could be construed as a potential conflict of interest.

## References

[B11] Al Khatib L. Obeid O. Sibai A. M. Batal M. Adra N. Hwalla N. (2006). Folate deficiency is associated with nutritional anaemia in Lebanese women of childbearing age. Public Health Nutr. 9 (7), 921–927. 10.1017/PHN2005921 17010258

[B51] Arora T. Backhed F. (2016). The gut microbiota and metabolic disease: current understanding and future perspectives. J. Intern. Med. 280 (4), 339–349. 10.1111/joim.12508 27071815

[B16] Balamurugan R. Mary R. R. Chittaranjan S. Jancy H. Shobana Devi R. Ramakrishna B. S. (2010). Low levels of faecal lactobacilli in women with iron-deficiency anaemia in south India. Br. J. Nutr. 104 (7), 931–934. 10.1017/s0007114510001637 20447323

[B35] Bentley M. E. Griffiths P. L. (2003). The burden of anemia among women in India. J. Eur. J. Clin. Nutr. 57 (1), 52–60. 10.1038/sj.ejcn.1601504 12548297

[B23] Callahan B. J. McMurdie P. J. Rosen M. J. Han A. W. Johnson A. J. Holmes S. P. (2016). DADA2: High-resolution sample inference from Illumina amplicon data. Nat. Methods 13 (7), 581–583. 10.1038/nmeth.3869 27214047 PMC4927377

[B53] Conlon M. A. Bird A. R. (2014). The impact of diet and lifestyle on gut microbiota and human health. Nutrients 7 (1), 17–44. 10.3390/nu7010017 25545101 PMC4303825

[B15] Cot M. le Hesran J. Y. Miailhes P. Roisin A. Fievet N. Barro D.. (1998). Effect of chloroquine prophylaxis during pregnancy on maternal haematocrit. Ann. Trop. Med. Parasitol. 92 (1), 37–43. 10.1080/00034989860157 9614452

[B33] Curtis H. Dirk G. Rob K. Sahar A. Jonathan H. B. Asif T. C.. (2012). Structure, function and diversity of the healthy human microbiome. Nature 486 (7402), 207–214. 10.1038/nature11234 22699609 PMC3564958

[B47] Cushing K. Alvarado D. M. Ciorba M. A. (2015). Butyrate and Mucosal Inflammation: New Scientific Evidence Supports Clinical Observation. Clin. Transl. Gastroenterol. 6, e108. 10.1038/ctg.2015.34 26312412 PMC4816278

[B10] de Benoist B. (2008). Conclusions of a WHO Technical Consultation on folate and vitamin B12 deficiencies. Food Nutr. Bull. 29 (2 Suppl), S238–S244. 10.1177/15648265080292s129 18709899

[B41] De Palma G. Nadal I. Medina M. Donat E. Ribes-Koninckx C. Calabuig M.. (2010). Intestinal dysbiosis and reduced immunoglobulin-coated bacteria associated with coeliac disease in children. BMC Microbiol. 10, 63. 10.1186/1471-2180-10-63 PMC2843610 20181275

[B24] DeSantis T. Z. Hugenholtz P. Larsen N. Rojas M. Brodie E. L. Keller K.. (2006). Greengenes, a chimera-checked 16S rRNA gene database and workbench compatible with ARB. Appl. Environ. Microbiol. 72 (7), 5069–5072. 10.1128/AEM.03006-05 16820507 PMC1489311

[B25] Dixon P. (2003). VEGAN, a package of R functions for community ecology. J. Veg. Sci. 14, 927–930. 10.1111/j.1654-1103.2003.tb02228.x

[B32] Giloteaux L. Goodrich J. K. Walters W. A. Levine S. M. Ley R. E. Hanson M. R. (2016). Reduced diversity and altered composition of the gut microbiome in individuals with myalgic encephalomyelitis/chronic fatigue syndrome. Microbiome 4 (1), 30. 10.1186/s40168-016-0171-4 27338587 PMC4918027

[B46] Gophna U. Konikoff T. Nielsen H. B. (2017). Oscillospira and related bacteria - From metagenomic species to metabolic features. Environ. Microbiol. 19 (3), 835–841. 10.1111/1462-2920.13658 28028921

[B19] Jaeggi T. Kortman G. A. Moretti D. Chassard C. Holding P. Dostal A.. (2015). Iron fortification adversely affects the gut microbiome, increases pathogen abundance and induces intestinal inflammation in Kenyan infants. Gut 64 (5), 731–742. 10.1136/gutjnl-2014-307720 25143342

[B49] Karlsson F. H. Tremaroli V. Nookaew I. Bergstrom G. Behre C. J. Fagerberg B.. (2013). Gut metagenome in European women with normal, impaired and diabetic glucose control. Nature 498 (7452), 99–103. 10.1038/nature12198 23719380

[B2] Kassebaum N. J. Jasrasaria R. Naghavi M. Wulf S. K. Johns N. Lozano R.. (2014). A systematic analysis of global anemia burden from 1990 to 2010. Blood 123 (5), 615–624. 10.1182/blood-2013-06-508325 24297872 PMC3907750

[B6] Katz J. Christian P. Dominici F. Zeger S. L. (2006). Treatment effects of maternal micronutrient supplementation vary by percentiles of the birth weight distribution in rural Nepal. J. Nutr. 136 (5), 1389–1394. 10.1093/jn/136.5.1389 16614435

[B18] Khosravi A. Yanez A. Price J. G. Chow A. Merad M. Goodridge H. S.. (2014). Gut microbiota promote hematopoiesis to control bacterial infection. Cell Host Microbe 15 (3), 374–381. 10.1016/j.chom.2014.02.006 24629343 PMC4144825

[B34] Koliada A. Syzenko G. Moseiko V. Budovska L. Puchkov K. Perederiy V.. (2017). Association between body mass index and Firmicutes/Bacteroidetes ratio in an adult Ukrainian population. BMC Microbiol. 17 (1), 120. 10.1186/s12866-017-1027-1 28532414 PMC5440985

[B42] Konikoff T. Gophna U. (2016). Oscillospira: a Central, Enigmatic Component of the Human Gut Microbiota. Trends Microbiol. 24 (7), 523–524. 10.1016/j.tim.2016.02.015 26996766

[B28] Koren O. Goodrich J. K. Cullender T. C. Spor A. Laitinen K. Bäckhed H. K.. (2012). Host remodeling of the gut microbiome and metabolic changes during pregnancy. Cell 150 (3), 470–480. 10.1016/j.cell.2012.07.008 22863002 PMC3505857

[B22] Kuczynski J. Stombaugh J. Walters W. A. Gonzalez A. Caporaso J. G. Knight R. (2012). Using QIIME to analyze 16S rRNA gene sequences from microbial communities. Curr. Protoc. Microbiol. 14, 927–930. 10.1002/9780471729259.mc01e05s27. Chapter 1, Unit 1E.5. PMC4477843 23184592

[B52] Kumar H. Du T. E. Kulkarni A. Aakko J. Linderborg K. M. Zhang Y.. (2016). Distinct Patterns in Human Milk Microbiota and Fatty Acid Profiles Across Specific Geographic Locations. Front. Microbiol. 7, e21313, 1619. 10.3389/fmicb.2016.01619 27790209 PMC5061857

[B21] Li J. L. Sheng H., Li Shao C. Zhi C. Z. Hong L. D. Cheng T.. (2019). Early-onset preeclampsia is associated with gut microbial alterations in antepartum and postpartum women. Front. Cell. Infect. Microbiol. 9, 224. 10.3389/fcimb.2019.00224 31297341 PMC6608563

[B7] Lee H. S. Kim M. S. Kim M. H. Kim Y. J. Kim W. Y. (2006). Iron status and its association with pregnancy outcome in Korean pregnant women. Eur. J. Clin. Nutr. 60 (9), 1130–1135. 10.1038/sj.ejcn.1602429 16639418

[B5] Ma A. G. Schouten E. Wang Y. Xu R. X. Zheng M. C. Li Y.. (2009). Anemia prevalence among pregnant women and birth weight in five areas in China. Med. Princ. Pract. 18 (5), 368–372. 10.1159/000226290 19648759

[B30] Manichanh C. Rigottier-Gois L. Bonnaud E. Gloux K. Pelletier E. Frangeul L.. (2006). Reduced diversity of faecal microbiota in Crohn’s disease revealed by a metagenomic approach. Gut 55 (2), 205–211. 10.1136/gut.2005.073817 16188921 PMC1856500

[B1] Milman N. (2011). Anemia–still a major health problem in many parts of the world! Ann. Hematol. 90 (4), 369–377. 10.1007/s00277-010-1144-5 21221586

[B39] Miquel S. Martin R. Rossi O. Bermudez-Humaran L. G. Chatel J. M. Sokol H.. (2013). Faecalibacterium prausnitzii and human intestinal health. Curr. Opin. Microbiol. 16 (3), 255–261. 10.1016/j.mib.2013.06.003 23831042

[B17] Muleviciene A. D’Amico F. Turroni S. Candela M. Jankauskiene A. (2018). Iron deficiency anemia-related gut microbiota dysbiosis in infants and young children: A pilot study. Acta Microbiol. Immunol. Hung 65 (4), 551–564. 10.1556/030.65.2018.045 30418043

[B29] Nuriel-Ohayon M. Neuman H. Ziv O. Belogolovski A. Barsheshet Y. Bloch N.. (2019). Progesterone Increases Bifidobacterium Relative Abundance during Late Pregnancy. Cell Rep. 27 (3), 730–736.e3. 10.1016/j.celrep.2019.03.075 30995472

[B13] Özsoylu Ş Aytekin M.N.J.A. (2011). Vitamin D deficiency and anemia. Front. Microbiol. 90, 6, 737–737. 10.1007/s00277-010-1078-y 20848103

[B55] Panda S. El khader I. Casellas F. Lopez Vivancos J. Garcia Cors M. Santiago A.. (2014). Short-term effect of antibiotics on human gut microbiota. PloS One 9 (4), e95476. 10.1371/journal.pone.0095476 24748167 PMC3991704

[B54] Paola M. D. Filippo C. D. Cavalieri D. Ramazzotti M. Poullet J. B. Massart S.. (2010). PP90 Impact Of Diet In Shaping Gut Microbiota Revealed By A Comparative Study In Children From Europe And Rural Africa. Proc. Natl. Acad. Sci. U.S.A. 107 (33), 14691–14696. 10.1073/pnas.1005963107 20679230 PMC2930426

[B12] Pinto A. V. Santos F. Almeida A. M. Cantuaria A. A. (1973). Trends of folate and vitamin B12 during pregnancy. Rev. Invest. Clin. 25 (2), 153–158. 4722230

[B8] Prema K. Neelakumari S. Ramalakshmi B. A. (1981). Anaemia and adverse obstetric outcome.

[B48] Qin J. Li Y. Cai Z. Li S. Zhu J. Zhang F.. (2012). A metagenome-wide association study of gut microbiota in type 2 diabetes. Nature 490 (7418), 55–60. 10.1038/nature11450 23023125

[B27] Rahmati S. Azami M. Badfar G. Parizad N. Sayehmiri K. (2020). The relationship between maternal anemia during pregnancy with preterm birth: a systematic review and meta-analysis. J. Matern. Fetal Neonatal. Med. 33 (15), 2679–2689. 10.1080/14767058.2018.1555811 30522368

[B43] Santoru M. L. Piras C. Murgia A. Palmas V. Camboni T. Liggi S.. (2017). Cross sectional evaluation of the gut-microbiome metabolome axis in an Italian cohort of IBD patients. Sci. Rep. 7 (1), 9523. 10.1038/s41598-017-10034-5 28842640 PMC5573342

[B14] Shulman C. E. Graham W. J. Jilo H. Lowe B. S. New L. Obiero J.. (1996). Malaria is an important cause of anaemia in primigravidae: evidence from a district hospital in coastal Kenya. Trans. R Soc. Trop. Med. Hyg. 90 (5), 535–539. 10.1016/s0035-9203(96)90312-0 8944266

[B26] Smith C. Teng F. Branch E. Chu S. Joseph K. S. (2019). Maternal and Perinatal Morbidity and Mortality Associated With Anemia in Pregnancy. Obstet. Gynecol. 134 (6), 1234–1244. 10.1097/aog.0000000000003557 31764734 PMC6882541

[B36] Sobhani I. Tap J. Roudot-Thoraval F. Roperch J. P. Letulle S. Langella P.. (2011). Microbial dysbiosis in colorectal cancer (CRC) patients. PloS One 6 (1), e16393. 10.1371/journal.pone.0016393 21297998 PMC3029306

[B40] Sokol H. Pigneur B. Watterlot L. Lakhdari O. Bermudez-Humaran L. G. Gratadoux J. J.. (2008). Faecalibacterium prausnitzii is an anti-inflammatory commensal bacterium identified by gut microbiota analysis of Crohn disease patients. Proc. Natl. Acad. Sci. U.S.A. 105 (43), 16731–16736. 10.1073/pnas.0804812105 18936492 PMC2575488

[B9] Starrs A. J. W. D. (2015). Preventing the Tragedy of Maternal Deaths. Front. Microbiol. 19, 5, 312. 10.2307/1966803

[B38] Swidsinski A. Loening-Baucke V. Verstraelen H. Osowska S. Doerffel Y. (2008). Biostructure of fecal microbiota in healthy subjects and patients with chronic idiopathic diarrhea. Gastroenterology 135 (2), 568–579. 10.1053/j.gastro.2008.04.017 18570896

[B45] Takahashi K. Nishida A. Fujimoto T. Fujii M. Shioya M. Imaeda H.. (2016). Reduced Abundance of Butyrate-Producing Bacteria Species in the Fecal Microbial Community in Crohn’s Disease. Digestion 93 (1), 59–65. 10.1159/000441768 26789999

[B44] Vesterbacka J. Rivera J. Noyan K. Parera M. Neogi U. Calle M.. (2017). Richer gut microbiota with distinct metabolic profile in HIV infected Elite Controllers. Sci. Rep. 7 (1), 6269. 10.1038/s41598-017-06675-1 28740260 PMC5524949

[B50] Vrieze A. Out C. Fuentes S. Jonker L. Reuling I. Kootte R. S.. (2014). Impact of oral vancomycin on gut microbiota, bile acid metabolism, and insulin sensitivity. J. Hepatol. 60 (4), 824–831. 10.1016/j.jhep.2013.11.034 24316517

[B3] W. H. Organization (1992). The prevalence of anaemia in women: a tabulation of available information (Geneva: World Health Organization).

[B4] World Health Organization (2019). Assessment for nutrition-related disorders in women during pregnancy. Available at: https://www.who.int/elena/titles/assessment-methods-pregnancy/en/.

[B31] Wang M. Karlsson C. Olsson C. Adlerberth I. Wold A. E. Strachan D. P.. (2008). Reduced diversity in the early fecal microbiota of infants with atopic eczema. J. Allergy Clin. Immunol. 121 (1), 129–134. 10.1016/j.jaci.2007.09.011 18028995

[B20] World Medical Association Declaration of Helsinki (2014). ethical principles for medical research involving human subjects. J. Am. Coll. Dent. 81 (3), 14–18. 10.1515/jwiet-2014-0117 25951678

[B37] Wu Z. W. Ling Z. X. Lu H. F. Zuo J. Sheng J. F. Zheng S. S.. (2012). Changes of gut bacteria and immune parameters in liver transplant recipients. Hepatobiliary Pancreat Dis. Int. 11 (1), 40–50. 10.1016/S1499-3872(11)60124-0 22251469

