# Supplementary material for: Gut Microbiota Signatures in Gestational Anemia
Source: Front Cell Infect Microbiol. 2021 Feb 25;11:549678. doi: 10.3389/fcimb.2021.549678 (PMC7947918; doi:10.3389/fcimb.2021.549678)
Supplement: Supplementary file 1 [file DataSheet_1.docx]

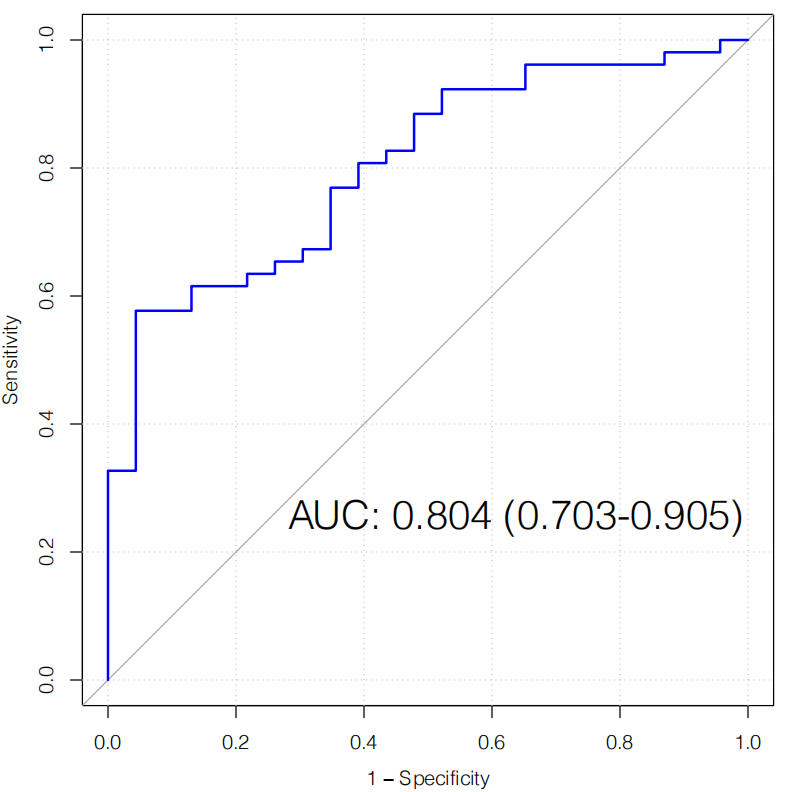

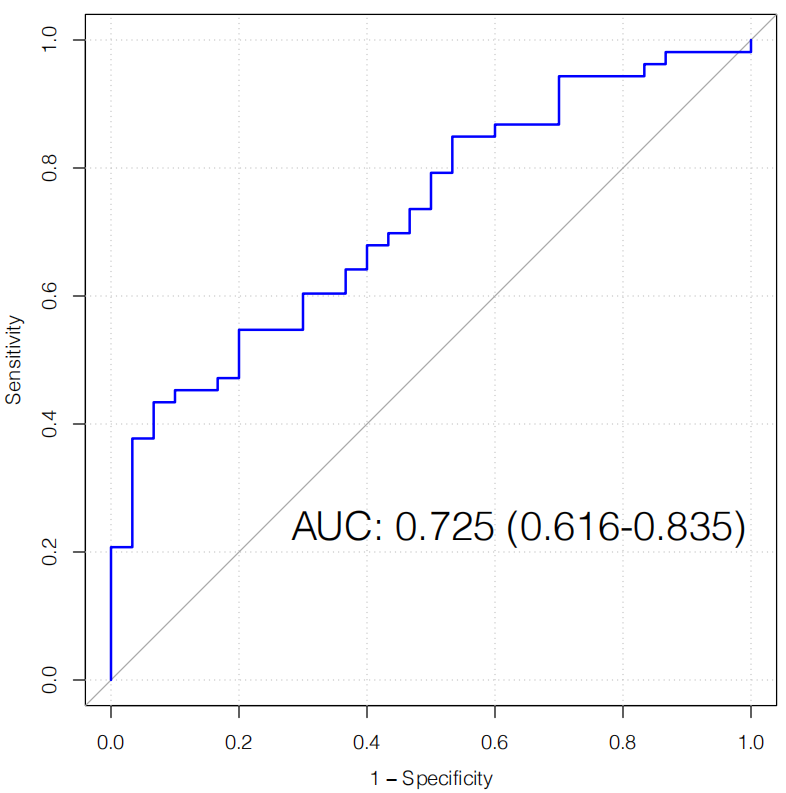


Figure S1. ROC curve of RF classification using significantly different OTUs combining with 8 clinical indicators. a) ROC curve of anemia classification in the first trimester; b) ROC curve of anemia classification in the third trimester.


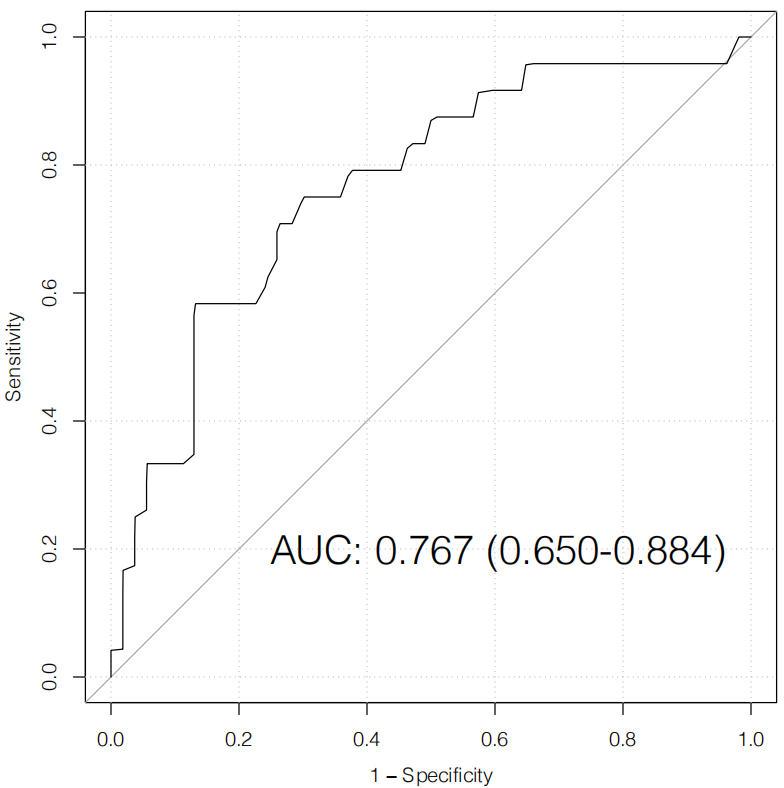

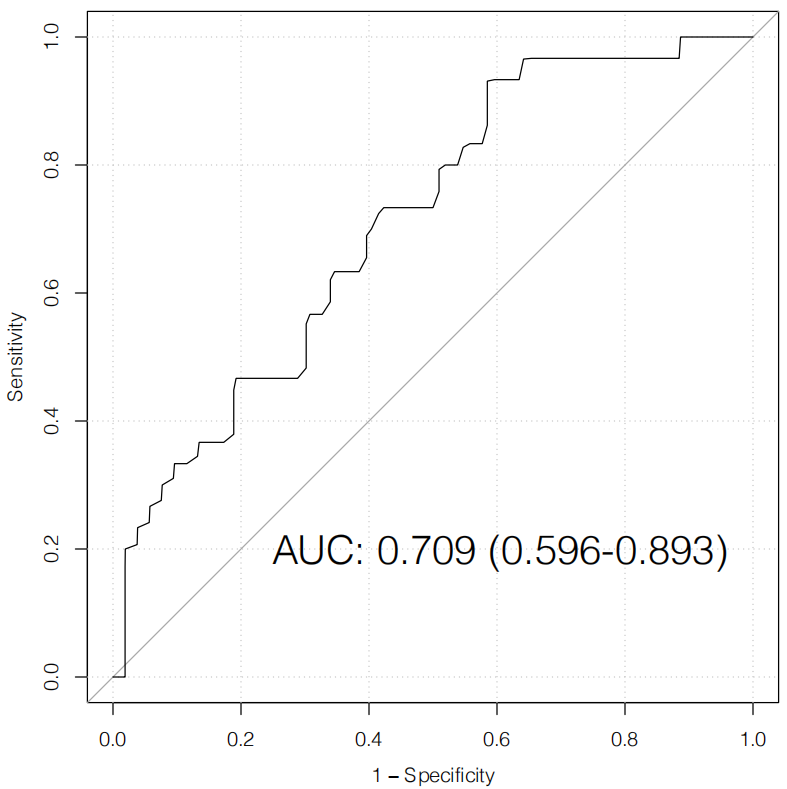


Figure S2. ROC curve of RF classification using significantly different OTUs based on OTUs selected by significantly high importance. a) ROC curve of anemia classification in the first trimester; b) ROC curve of anemia classification in the third trimester.


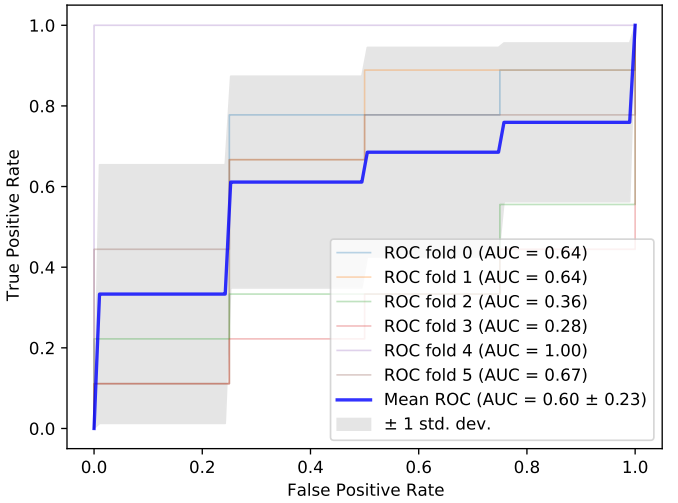

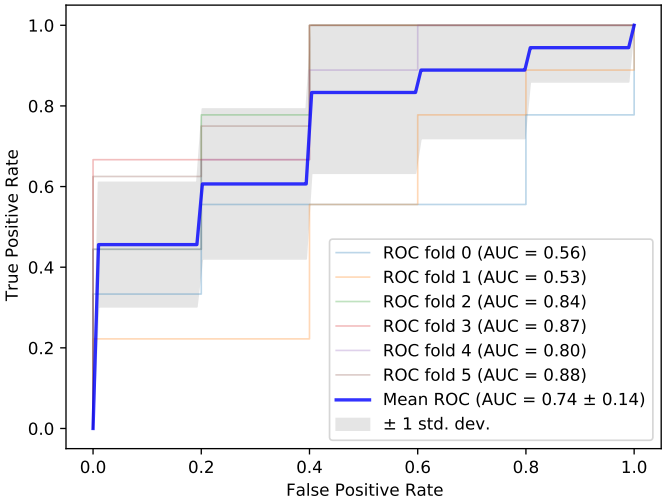


Fig S3. ROC curve of SVM classification using significantly different OTUs combining with 8 clinical indicators. a) ROC curve of anemia classification in the first trimester; b) ROC curve of anemia classification in the third trimester.


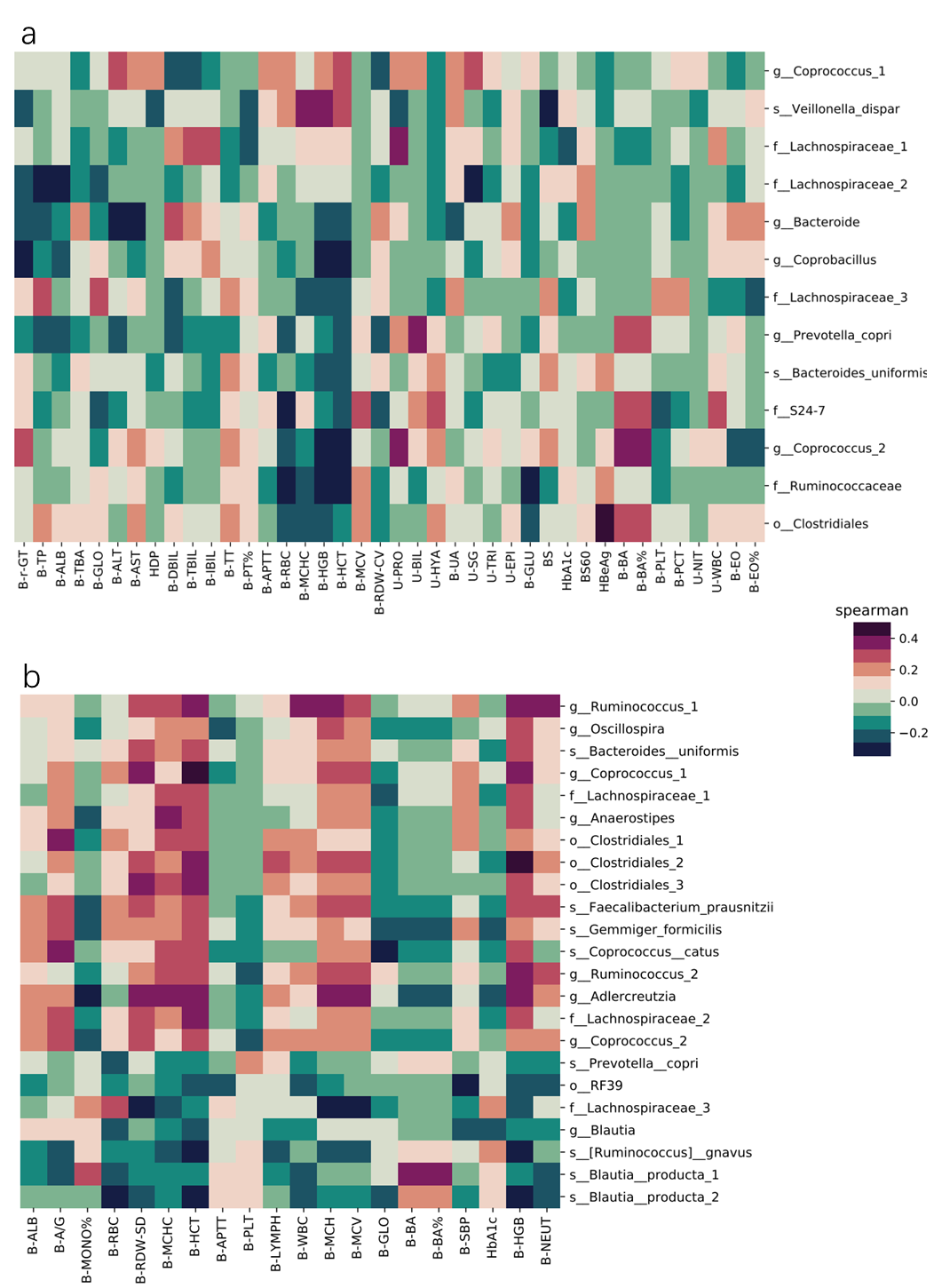


Fig S4. Correlation between GA-associated OTUs and the clinical parameters. a) The heatmap panel shows the Spearman correlation coefficient between the GA- associated OTUs in the first trimester and clinical parameter. b) The heatmap panel shows the Spearman correlation coefficient between the GA- associated OTUs in the third trimester and clinical parameter.
